# Favipiravir and Ribavirin Inhibit Replication of Asian and African Strains of Zika Virus in Different Cell Models

**DOI:** 10.3390/v10020072

**Published:** 2018-02-09

**Authors:** Ji-Ae Kim, Rak-Kyun Seong, Mukesh Kumar, Ok Sarah Shin

**Affiliations:** 1 Department of Biomedical Sciences, College of Medicine, Korea University Guro Hospital, Seoul 08308, Korea; kja0910kr@gmail.com (J.-A.K.); cyeano66@naver.com (R.-K.S.); 2 Department of Tropical Medicine, Medical Microbiology and Pharmacology, Pacific Center for Emerging Infectious Diseases Research, John A. Burns School of Medicine, University of Hawaii at Manoa, Honolulu, HI 96813, USA

**Keywords:** favipiravir, ribavirin, Zika virus, hNPCs, anti-viral

## Abstract

Zika virus (ZIKV) has recently emerged as a new public health threat. ZIKV infections have caused a wide spectrum of neurological diseases, such as Guillain–Barré syndrome, myelitis, meningoencephalitis, and congenital microcephaly. No effective therapies currently exist for treating patients infected with ZIKV. Herein, we evaluated the anti-viral activity of favipiravir (T-705) and ribavirin against Asian and African strains of ZIKV using different cell models, including human neuronal progenitor cells (hNPCs), human dermal fibroblasts (HDFs), human lung adenocarcinoma cells (A549) and Vero cells. Cells were treated with favipiravir or ribavirin and effects on ZIKV replication were determined using quantitative real-time PCR and plaque assay. Our results demonstrate that favipiravir or ribavirin treatment significantly inhibited ZIKV replication in a dose-dependent manner. Moreover, favipiravir treatment of ZIKV-infected hNPCs led to reduced cell death, enhanced AKT pathway phosphorylation, and increased expression of anti-apoptotic factor B cell lymphoma 2. In conclusion, our results demonstrate conclusively that favipiravir inhibits ZIKV replication and prevents cell death, and can be a promising intervention for ZIKV-associated disease.

## 1. Introduction

Zika virus (ZIKV) was originally isolated in 1947 from a febrile rhesus monkey in the Zika forest in Uganda [1]. Phylogenetic analysis of ZIKV genomes reveals African and Asian strains of ZIKV as two distinct lineages [2,3]. The African lineage viruses have caused sporadic human infections over the last century, resulting in mild, febrile disease symptoms. The Asian lineage has however emerged at a larger scale displaying vector-borne as well as human-to-human transmission, causing neurological disease. Infection by Asian lineage ZIKVs has been linked to the development of severe fetal abnormalities that include spontaneous abortion, stillbirth, hydranencephaly, microcephaly, and placental insufficiency that may cause intrauterine growth restriction. An increased incidence of Guillain–Barre syndrome (GSB), neuropathy of the peripheral nervous system, has also been reported in ZIKV-infected patients [4,5,6,7,8,9]. Despite the global threat of ZIKV outbreaks, there is no licensed vaccine or specific anti-viral therapy to prevent or treat infections by ZIKV.

ZIKV is an RNA virus that is closely related to other flaviviruses including yellow fever virus, dengue viruses, Japanese encephalitis virus, and West Nile virus [10]. The ZIKV genome is comprised of a single-stranded, positive-sense 11-kb RNA that contains three structural and seven nonstructural genes. After binding and entry into a cell, viral RNA is released into the cytoplasm and translated in the endoplasmic reticulum into a single polyprotein. Viral and host proteases cleave this polyprotein into three structural (capsid, membrane [M], and envelope [E]) and seven nonstructural (NS1, NS2A, NS2B, NS3, NS4A, NS4B, and NS5) proteins [11]. The NS proteins of ZIKV are known to contribute to an evasion strategy that suppresses host innate immune defenses, allowing successful establishment of a productive infection [12]. *NS5* encodes the RNA-dependent RNA polymerase (RdRp) which is required for viral RNA synthesis. Therefore, targeting RdRp and its enzymatic activities may be a promising intervention to inhibit ZIKV replication.

Favipiravir (T-705) is a novel antiviral compound that selectively and potently inhibits the RdRp common to several RNA viruses, including influenza virus [13,14,15]. Ribavirin is a guanosine analogue that has broad-spectrum activity against several RNA and DNA viruses [16]. Although originally approved only for the treatment of severe respiratory syncytial virus infection in children, ribavirin has been used in the treatment of Lassa fever virus infection, influenza A and B, hepatitis C and other viruses [17]. In this study, we evaluated the anti-viral effects of favipiravir and ribavirin on ZIKV infection in different cell types, including human neuronal progenitor cells (hNPCs), human dermal fibroblasts (HDFs), human lung adenocarcinoma cells (A549), and Vero cells. Dermal fibroblasts are one of the first cell types exposed to ZIKV during a blood meal by an infected mosquito. ZIKV has been shown to replicate and induce cell death in neuronal cells of fetal mice as well as in human neural progenitor cells and brain organoids, a mechanism thought to play an important role in the pathogenesis of ZIKV-associated disease. To broadly define the anti-viral function of favipiravir and ribavirin, we also used A549 cells and Vero cells. We assessed the anti-viral activity of favipiravir and ribavirin against both Asian and African ZIKV strains. In addition, we examined ZIKV-induced neuronal cell death and modulation of cell growth and apoptosis signaling in the presence of favipiravir and ribavirin. In conclusion, our results demonstrate conclusively that favipiravir had robust anti-viral activity in various cells types and protects against ZIKV-mediated cell death in hNPCs.

## 2. Materials and Methods

### 2.1. Cells, Viruses, and Reagents

Human A549 lung adenocarcinoma cells and Vero kidney epithelial cells were obtained from the American Type Culture Collection (ATCC; Manassas, VA, USA). A549 cells were cultured at 37 °C in RPMI 1640 medium (Corning Mediatech, Corning, NY, USA) supplemented with 10% fetal bovine serum (FBS), 100 U/mL penicillin, and 100 μg/mL streptomycin. Vero cells were cultured at 37 °C in DMEM supplemented with 10% FBS, 100 U/mL penicillin, and 100 μg/mL streptomycin. Human dermal fibroblasts (HDFs) (Lonza, Basel, Switzerland) were grown as adherent cultures in fibroblast basal medium supplemented with fibroblast growth media (FGM) SingleQuots (Lonza) and cultured as described previously [18].

For the generation of hNPCs, human embryonic stem cells (hESCs) were grown under standard culture conditions with a feeder layer and then transferred to a matrigel-coated plate with mouse embryonic fibroblasts (MEFs) conditioned media and maintained for two consecutive passages. To induce neural differentiation, hESC media was replaced with DMEM/F12 supplemented with 2% B27, 100 ng/mL fibroblast growth factor, 100 ng/mL epidermal growth factor and 5 μg/mL heparin. Partially differentiated hESCs were dissociated with accutase and plated on geltrex-coated plates. Homogenous populations of NPCs were obtained after three continuous passages. Matrigel was purchased from BD Biosciences, San Jose, CA, USA and other reagents were purchased from Invitrogen, Carlsbad, CA, USA.

ZIKV MR766 (African lineage), PRVABC59 (Asian lineage) and P6-740 (Asian lineage) strains were purchased from the ATCC and propagated in Vero cells. Viral titers were determined using a standard plaque assay as described previously [19]. Favipiravir was purchased from AdooQ Bioscience, Irvine, CA, USA and ribavirin was purchased from Sigma-Aldrich, St. Louis, MI, USA.

### 2.2. ZIKV Infection and Drug Treatment

Cells were treated with drug (favipiravir or ribavirin) prior to infection overnight. During infection, drug was removed and was introduced immediately after infection until sampling (24 h or 72 h). Cells were infected with ZIKV, and treated with either DMSO control (0 M), favipiravir (1, 10, 25, and 50 μM), or ribavirin (1, 10, 25, and 50 μg/mL) until sampling.

### 2.3. MTT Assay

Cells were seeded in 96-well plates. After 24 h, the media were changed and various concentrations of favipiravir or ribavirin were added. The incubation continued for 24 h at 37 °C. After the incubation, 3-(4,5-dimethylthiazolyl-2)-2,5-diphenyltetrazolium bromide (MTT) (Sigma-Aldrich) solution was added for 4 h. The optical density at 570 nm was then measured using a spectrophotometer as described previously [20].

### 2.4. Confocal Microscopy

Cells were seeded onto coverslips in 24-well plates and treated with either favipiravir or ribavirin, followed by ZIKV MR766 infection at various time points. Cells were washed with PBS, fixed with 4% paraformaldehyde (PFA) and permeablized with 0.1% Triton X-100 as described previously [21,22]. Cells were then stained with anti-pan-flavivirus envelope (E) monoclonal antibody (1:200 dilution; Abcam, Cambridge, UK), followed by anti-mouse Alexa 594 conjugated antibody (Invitrogen). Coverslips were mounted on glass slides using mounting media containing 4,6-diamidino-2-phenylindole (DAPI) and were examined using a confocal microscope (LSM700; Carl Zeiss, Oberkochen, Germany).

### 2.5. Quantitative Real-Time PCR (qRT-PCR)

Total RNA (0.5 μg, isolated with Trizol reagent (Invitrogen) was reverse transcribed to generate cDNA using a RT system (Promega, Madison, WI, USA) for 1 h at 42 °C. The resulting cDNA was used as a template for qRT-PCR quantification of ZIKV transcript levels using a Power SYBR Green PCR Master Mix (Thermo Fisher Scientific, Waltham, MA, USA). ZIKV primer sequences used have been reported previously [19,23,24]. The cycling parameters were 95 °C for 15 min, followed by 40 cycles of 30 s at 95 °C and 1 min at 60 °C. Glyceraldehyde 3-phosphate dehydrogenase (GAPDH) mRNA was used as a normalizing control.

### 2.6. Western Blotting

Cells were lysed at the specified time points using RIPA buffer (Sigma-Aldrich). Lysates were separated by sodium dodecyl sulfate polyacrylamide gel electrophoresis (SDS-PAGE) on 10–12% acrylamide gels. Proteins were transferred to polyvinylidene difluoride (PVDF) membranes and blocked with 5% (*w*/*v*) skim milk in Tris-buffered saline (0.2 M Tris, 1.36 M NaCl) supplemented with 0.1% (*v*/*v*) Tween-20 (TBS-Tw) for 1 h at 25 °C as described previously [25]. This was followed by an overnight incubation with primary antibodies (Cell Signaling Technologies, Danvers, MA, USA) raised against phospho-AKT and total AKT at 4 °C. After three washes in TBS/Tween-20, the membranes were incubated with HRP-conjugated anti-rabbit or anti-mouse IgG secondary antibodies for 1 h at 25 °C. Membranes were washed with TBS/Tween-20, incubated with Western Lumi Pico solution (ECL solution kit; DoGen, Seoul, Korea). Signals were determined using a Fusion Solo Imaging System (Vilber Lourmat, Collégien, France). Band intensities were quantified by Fusion-Capt analysis software (Vilber Lourmat, Collégien, France).

### 2.7. Statistical Analysis

Quantitative data were expressed as means ± standard error of the mean (SEM). Statistical analysis was performed using Graphpad Prism (Graphpad Software, La Jolla, CA, USA) by comparing controls to treated groups. Student’s t tests were performed to compare individual treatments.

## 3. Results

### 3.1. Favipiravir and Ribavirin Inhibit ZIKV Infection in HDFs, A549 and Vero Cells

**Figure 1:**
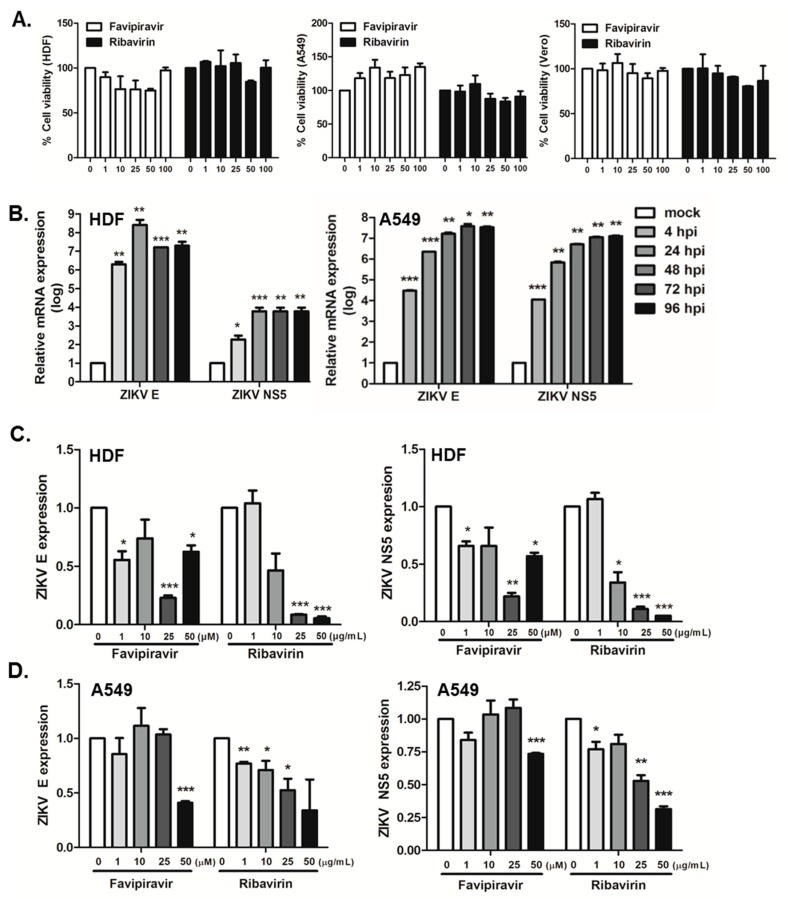
Zika virus (ZIKV) replication efficiency in favipiravir and ribavirin-treated human dermal fibroblasts (HDFs) and A549 cells. (**A**) HDFs, A549, and Vero cells were treated with various doses of favipiravir (μM), or ribavirin (μg/mL) and incubated for 24 h. At the end of incubation, cell viability was determined using MTT assays. Each value represents the mean ± SEM (*n* = 4). (**B**) HDFs and A549 cells were infected with ZIKV at MOI of 1 for indicated times. ZIKV E and NS5 expressions are shown. * *p* < 0.05; ** *p* < 0.01; *** *p* < 0.001, compared with mock control. (**C**) HDFs and (**D**) A549 cells were infected with ZIKV at MOI of 1 and treated with either a DMSO control (0 μM), favipiravir (1, 10, 25, and 50 μM), or ribavirin (1, 10, 25, and 50 μg/mL) for 24 h. qRT-PCR was performed to measure ZIKV E and NS5 mRNA levels. The expression of viral transcripts was calculated in relation to the expression level of GAPDH mRNA and expressed as fold-changes relative to expression levels in DMSO control-treated cells. * *p* < 0.05; ** *p* < 0.01; *** *p* < 0.001, compared with DMSO control-treated cells.

We first evaluated the cytotoxic effects of favipiravir or ribavirin using 3-(4,5-dimethyl-2-thiazolyl)-2, 5-diphenyl-2H-tetrazolium bromide (MTT) assay. Cell viability was compared to vehicle-treated (DMSO) controls. As depicted in Figure 1A, favipiravir and ribavirin did not impact cell viability at concentrations of 100 μM or lower in all three cell types.

We then evaluated the replication kinetics of ZIKV in HDFs and A549 cells. Cells were infected with ZIKV (African MR766 strain) at MOI of 1, and ZIKV envelope (*E*) and nonstructural protein 5 (*NS5*) gene expression was measured by qRT-PCR. We demonstrate that both HDFs and A549 cells are highly susceptible to ZIKV infection (Figure 1B). We next investigated the effect of favipiravir and ribavirin treatment on ZIKV gene expression. Cells were infected with ZIKV and treated with either favipiravir or ribavirin at various concentrations for 24 h. Favipiravir treatment significantly inhibited ZIKV replication in a dose-dependent manner. Favipiravir treatment at a concentration of 25 μM resulted in > 75% reduction in the expression of ZIKV *E* and *NS5* transcripts in HDFs (Figure 1C), whereas 50 μM favipiravir in A549 cells was required for a 67% and 26% decrease in ZIKV *E* and *NS5* expression, respectively (Figure 1D). Ribavirin also resulted in a significant suppression in ZIKV gene expression.

**Figure 2:**
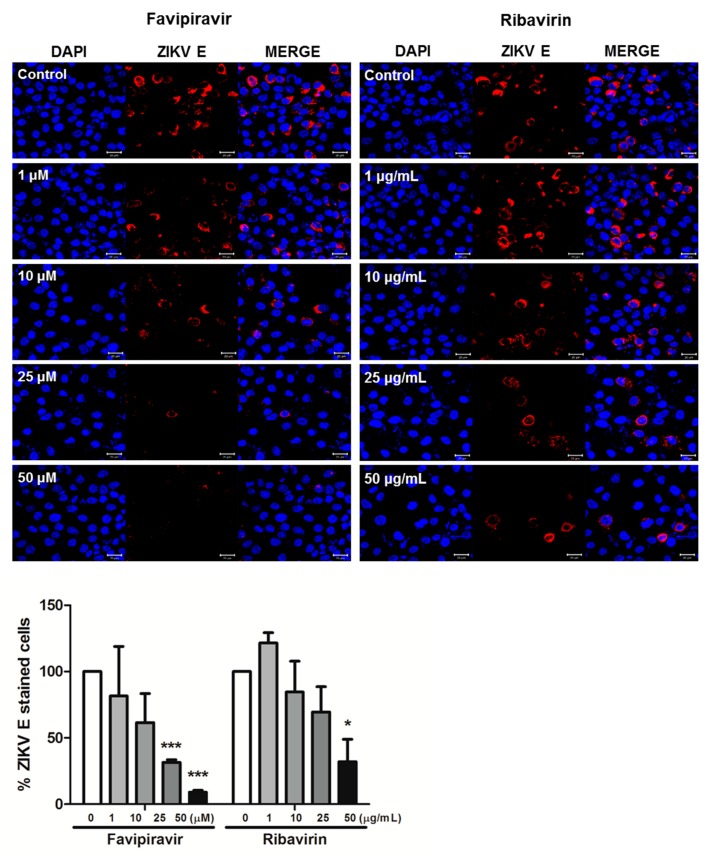
Favipiravir or ribavirin treatment resulted in reduced ZIKV E protein expression. A549 cells were infected with ZIKV (MOI-5) and treated with either DMSO control (0 M), favipiravir (1, 10, 25, and 50 μM), or ribavirin (1, 10, 25, and 50 μg/mL) for 72 h post infection. The ZIKV envelope (E) protein was immunostained with an anti-pan-flavivirus envelope monoclonal antibody. ZIKV E is indicated in red and cell nuclei are stained blue. The images are representative of three independent experiments. Magnification shown is 400 × and the scale bar represents 20 μm. % ZIKV E-stained cells were calculated and the graph represents an average of three independent experiments. * *p* < 0.05; *** *p* < 0.001.

**Figure 3:**
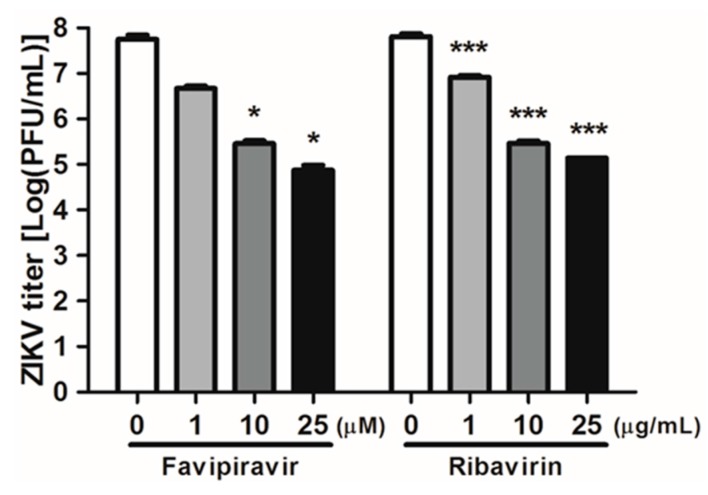
ZIKV infectivity titers following treatment with favipiravir and ribavirin. Vero cells were infected with ZIKV at MOI of 1 and treated with either a DMSO control (0 μM), favipiravir (1, 10, 25, and 50 μM), or ribavirin (1, 10, 25, and 50 μg/mL) for 24 h. Viral replication was determined using plaque assay on cultured supernatants of ZIKV-infected Vero cells. Data are representative of three independent experiments, and each was performed in duplicate. Error bars represent the standard deviation of three biological replicates. * *p* < 0.05; *** *p* < 0.001, compared with DMSO control-treated cells.

We next measured the expression of ZIKV E protein using confocal microscopy in A549 cells. Cells were infected with ZIKV and treated with either favipiravir or ribavirin at various concentrations for 72 h. Immunofluorescence staining corroborated the qRT-PCR results (Figure 1D) as favipiravir or ribavirin treatment decreased the expression of ZIKV E protein (Figure 2). We further analyzed the effect of favipiravir and ribavirin on ZIKV infection in Vero cells. Viral replication was determined using plaque assay on cultured supernatants of ZIKV-infected Vero cells. The number of plaques decreased significantly in favipiravir or ribavirin treated cells in a dose-dependent manner (Figure 3). Taken together, these results showed that favipiravir or ribavirin treatment results in significant reduction in expression of ZIKV-specific *E* and *NS5* genes, E protein, and production of infectious virus particles. Moreover, our data also indicate that ribavirin’s inhibitory capacity on ZIKV replication is better than that of favipiravir.

### 3.2. Favipiravir and Ribavirin Inhibit ZIKV Infection in hNPCs

**Figure 4:**
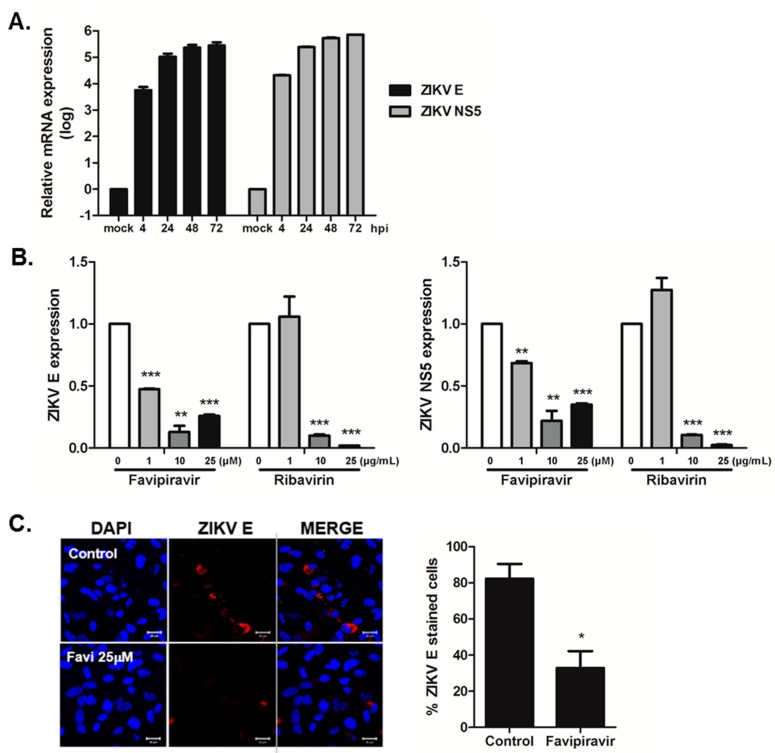
Anti-viral activity of favipiravir and ribavirin in hNPCs. (**A**) hNPCs were infected with ZIKV at MOI of 10 for indicated times. ZIKV E and NS5 expressions are shown. (**B**) hNPCs were infected with ZIKV (MOI-10) and treated with either DMSO control (0 M), favipiravir (1, 10, 25 μM), or ribavirin (1, 10, 25 μg/mL) for 72 h after infection, and the levels of ZIKV E and NS5 mRNA were measured over time using qRT-PCR. The expression of viral genes was normalized to GAPDH and the levels of expression for the control DMSO-treated group were arbitrarily set to 1. * *p* < 0.05; ** *p* < 0.01; *** *p* < 0.001, compared with DMSO control-treated cells. (**C**) Immunofluorescent staining of ZIKV E protein in hNSCs at 72 h post infection is shown. Magnification shown is 400 × and the scale bar represents 20 μm. % ZIKV E-stained cells were calculated and the graph shows an average of three independent experiments. * *p* < 0.05.

Recent studies have shown that ZIKV can infect neuronal progenitors, neurospheres, and organoids derived from human induced pluripotent stem cells (iPSCs) or embryonic stem cells [26,27,28]. To assess the anti-viral activity of favipiravir and ribavirin in hNPCs, we first generated human neuronal progenitor cells (hNPCs). Similar to previous studies, hNPCs were highly susceptible to ZIKV infection (Figure 4A). Then, we measured the transcript levels of ZIKV *E* and *NS5* in the presence of favipiravir or ribavirin. At 24 h post infection, there was a significant dose-dependent reduction in viral ZIKV *E* and *NS5* transcript levels, suggesting that favipiravir and ribavirin can suppress viral gene expression in hNPCs (Figure 4B). This was confirmed at the protein level by reduced ZIKV E staining in favipiravir-treated hNPCs at 72 h post infection (Figure 4C).

### 3.3. Favipiravir Inhibits Infection of Asian ZIKV Strains in hNPCs

**Figure 5:**
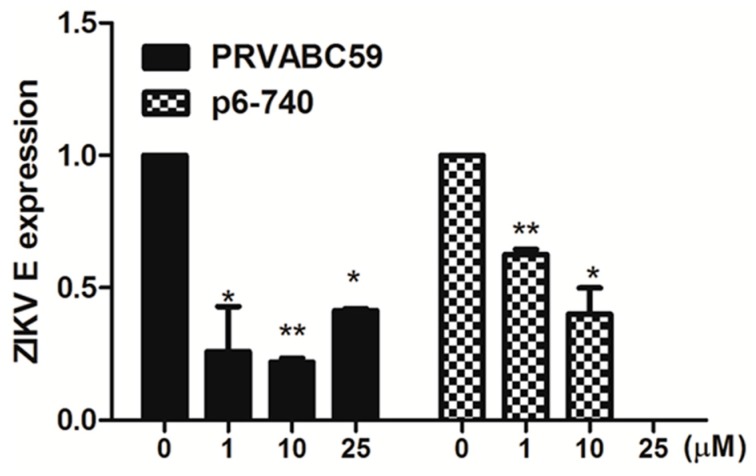
Favipiravir inhibits infection of Asian ZIKV strains in hNPCs. hNPCs were infected with ZIKV PRVABC59 or ZIKV P6-740) at MOI of 1, and treated with either DMSO control (0 μM), favipiravir (1, 10, 25 μM) for 72 h after infection. Levels of ZIKV E and NS5 mRNA were measured in PRVABC59 or P6-740 infected hNPCs over time using qRT-PCR. * *p* < 0.05; ** *p* < 0.01, compared with DMSO control-treated cells.

Asian and African ZIKV strains differ in their abilities to infect cells of the central nervous system and to cause cell death [29,30,31,32,33]. Asian lineage ZIKVs have been demonstrated to cause neurological disease in humans. To determine the inhibition spectrum of favipiravir against ZIKV infection, hNPCs were infected with two different ZIKV strains of Asian lineage (PRVABC59 and P6-740). As depicted in Figure 5, ZIKV gene expression was significantly reduced upon the treatment of favipiravir in PRVABC59 or P6-740-infected cells.

### 3.4. Favipiravir Prevents Cell Death in ZIKV-Infected hNPCs, and Modulates Cell Growth and Apoptosis Signaling

**Figure 6:**
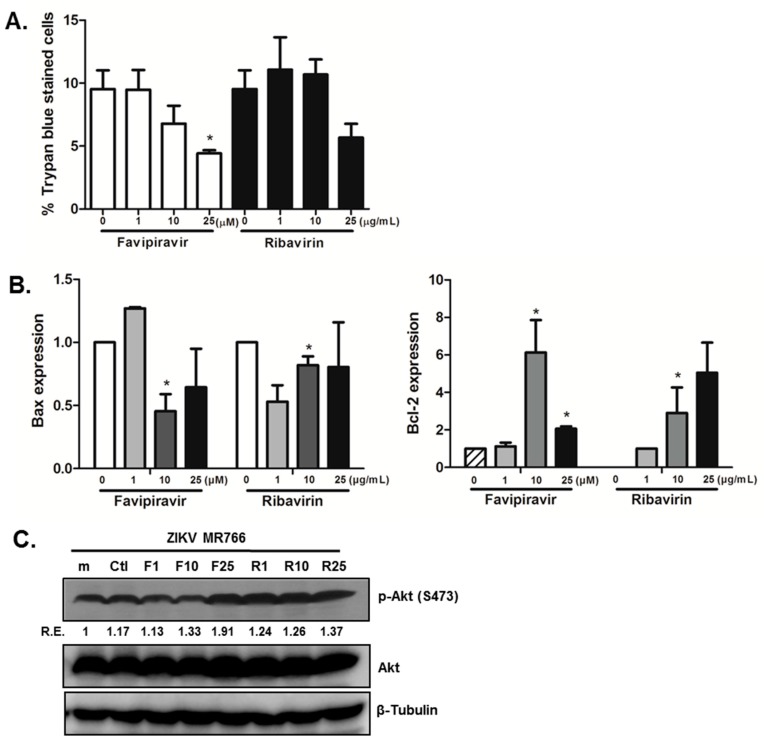
Favipiravir treatment in hNPCs results in the reduction of ZIKV-induced neuronal cell death. (**A**) Trypan blue staining was performed and the graph represents the proportion of cells with positive staining. The averages of three independent experiments are shown. * *p* < 0.05. (**B**) BCL2 and BAX mRNA expression was measured by qRT-PCR. The expression of target genes was normalized to GAPDH. Data are shown as the means ± SEM from three independent experiments. * *p* < 0.05 compared with DMSO control-treated cells. (**C**) Phosphorylation of AKT was detected by western blot analysis using a specific antibody, with normalized densitometric units plotted against treatment (shown as numbers). The representative image of three independent experiments is shown. m(mock), Ctl (DMSO control), F (favipiravir), R (ribavirin), R.E. (Relative Expression).

To characterize the effects of favipiravir on neuronal cell death, we compared the percentage of trypan blue-positive stained cells in control (DMSO treated) versus favipiravir or ribavirin treated ZIKV-infected cells. We demonstrate that treatment with 25 μM favipiravir resulted in a significantly decreased proportion of cells positive for trypan blue staining (Figure 6A). We next assessed whether this decrease in neuronal cell death involved modulation of cell growth or apoptosis signaling. We observed that the expression of the anti-apoptotic factor B cell lymphoma 2 (*BCL2*), and pro-apoptotic factor Bcl-2 associated X protein (*BAX*), changed following favipiravir and ribavirin treatment. There was a significant increase in *BCL2* mRNA levels, whereas *BAX* mRNA levels decreased after favipiravir and ribavirin treatment (Figure 6B). Furthermore, phosphorylation of AKT was increased in the presence of favipiravir compared to control-treated ZIKV-infected cells (Figure 6C). Therefore, it is likely that favipiravir treatment attenuates ZIKV-induced cell apoptosis in hNPCs via modulation of apoptosis-regulatory genes and promotes survival signaling via the PI3K/AKT pathway.

## 4. Discussion

In this study, we have characterized the in vitro anti-ZIKV activity of favipiravir and ribavirin by measuring the transcript levels of essential ZIKV genes, viral protein expression, and production of infectious virus particles. We assessed the anti-viral activity of favipiravir and ribavirin in various cell models, including hNPCs, HDFs, A549, and Vero cells. We found that favipiravir treatment resulted in a reduction in ZIKV-induced cell death, and promoted cell survival signal via phosphorylation of the PI3K/AKT pathway.

During the submission of our manuscript, several groups independently verified anti-ZIKV activities of favipiravir [34,35,36,37]. Baz et al. screened in vitro susceptibility of different strains of ZIKV to favipiravir and ribavirin. Both drugs showed anti-ZIKV activities against all the strains but there was no synergistic effect from the combination of two drugs. Another recent study by Pires de Mello et al.’s also demonstrated inhibitor effect of favipiravir on ZIKV infection in Vero cells. However, they used higher concentration of favipiravir to achieve similar inhibition as observed in our study. Pires de Mello et al.’s also reported the lack of synergistic activity of favipiravir and ribavirin in vitro. Although we did not test the effect of combination of two drugs, we observed that ribavirin’s inhibitory capacity on ZIKV replication appeared to be much better than that of favipiravir. Interestingly, Lanko et al. tested the effect of favipiravir in induced pluripotent stem cell (iPSC)-differentiated neuronal cells, including cortical neurons, motor neurons, and astrocytes and found that both MR766 and PRVABC59 strains of ZIKV replication was not affected by favipiravir. Our results are different from Lanko et al.’s study in that in our study, we examined the effects that favipiravir had on ZIKV infections of undifferentiated hNSCs, a major target cell type of the virus. It is still unknown why favipiravir or ribavirin did not significantly affect ZIKV replication in hiPSC-derived neuronal cells, but it is possible that cell type specific expression of ZIKV receptors or different immune defense mechanisms among these cells may account for the underlying mechanisms determining the anti-viral activity of favipiravir.

Many studies have shown that favipiravir has a potent therapeutic effect against a broad range of influenza viruses, including the highly pathogenic avian influenza virus (H5N1) and viruses resistant to neuraminidase inhibitors (NA) inhibitors [15,38,39]. A phase III clinical evaluation of favipiravir for anti-influenza therapy has been completed in Japan and approved for human use against influenza virus in Japan, whereas two phase II studies have been completed in the United States [13]. Favipiravir also inhibits the replication of many other RNA viruses, including members of the arenavirus, bunyavirus, and flaviviruses, such as YFV and WNV [14,40]. Several other approved drugs, such as sofosbuvir and suramin, have also proved to be effective against ZIKV [41,42,43,44,45]. It would therefore be interesting to investigate whether a combination of these drugs with favipiravir would provide improved antiviral efficacy against ZIKV infection.

Neurogenesis is a tightly regulated process in which neurons differentiate from neural stem cells (hNSCs) or hNPCs. This process is most active during pre-natal development and is responsible for brain development [46]. Microcephaly is caused by a developmental defect that associates with genetic mutations affecting neurogenesis and migration [47]. The cause of microcephaly is due to diverse factors, but the underlying biological mechanisms are highly likely to be affected by disruption of differentiation, and the dysregulation of neuronal cell proliferation and apoptosis. Several cellular pathways are critical for neurogenesis, including the PI3K/AKT pathway [48]. Liang et al. (2016) have suggested that ZIKV NS4A and NS4B can cooperatively suppress the AKT/mTOR pathway in hNSCs to inhibit neurogenesis [49]. In our study, we show that favipiravir treatment in hNSCs resulted in upregulation of AKT pathways, which has not been reported before. As several studies have shown that hNPCs are a major target cell type of ZIKV infection, they are an excellent model system to investigate the impact and mechanisms of ZIKV during human brain development. ZIKV infection can lead to cell cycle arrest, apoptosis, and inhibition of NPC differentiation, resulting in cortical thinning and microcephaly [50]. Bcl proteins are essential for the regulation of apoptosis, as mutations in this family interfere with normal programmed cell death. We examined if favipiravir treatment affected the expression of certain key Bcl transcripts, such as *BCL2* and *BAX*. BCL2 was the first anti-apoptotic regulator to be identified, while BAX is a pro-apoptotic family member [51]. Favipiravir treatment resulted in increased *BCL2* mRNA levels, but decreased levels of *BAX* mRNA, suggesting that favipiravir affects ZIKV-mediated cell death in hNPCs via modulation of Bcl family member expression. It will be interesting to further investigate the role of favipiravir in ZIKV-induced tissue damage in human cortical organoids and the embryonic brain of a ZIKV-induced mouse model of microcephaly. Of note, favipiravir has been described as a potent inducer of lethal mutagenesis in several studies [52,53,54,55]. Therefore, further studies to define the underlying mechanism of action of the favipiravir on ZIKV are warranted. 

In summary, our results indicate that favipiravir and ribavirin effectively suppressed viral replication of ZIKV, and ameliorated the ZIKV-mediated cell death of hNPCs. Further studies involving in vivo evaluation of favipiravir administration in animal models will be important to further assess the therapeutic effects of favipiravir.
